# Population-Based Analysis of Invasive Nontypeable Pneumococci Reveals That Most Have Defective Capsule Synthesis Genes

**DOI:** 10.1371/journal.pone.0097825

**Published:** 2014-05-15

**Authors:** In Ho Park, K. Aaron Geno, Logan K. Sherwood, Moon H. Nahm, Bernard Beall

**Affiliations:** 1 Department of Pathology, University of Alabama at Birmingham, Birmingham, Alabama, United States of America; 2 Respiratory Diseases Branch, Centers for Disease Control and Prevention, Atlanta, Georgia, United States of America; 3 Department of Microbiology, University of Alabama at Birmingham, Birmingham, Alabama, United States of America; Instituto Butantan, Brazil

## Abstract

Since nasopharyngeal carriage of pneumococcus precedes invasive pneumococcal disease, characteristics of carriage isolates could be incorrectly assumed to reflect those of invasive isolates. While most pneumococci express a capsular polysaccharide, nontypeable pneumococci are sometimes isolated. Carriage nontypeables tend to encode novel surface proteins in place of a capsular polysaccharide synthetic locus, the *cps* locus. In contrast, capsular polysaccharide is believed to be indispensable for invasive pneumococcal disease, and nontypeables from population-based invasive pneumococcal disease surveillance have not been extensively characterized. We received 14,328 invasive pneumococcal isolates through the Active Bacterial Core surveillance program during 2006–2009. Isolates that were nontypeable by Quellung serotyping were characterized by PCR serotyping, sequence analyses of the *cps* locus, and multilocus sequence typing. Eighty-eight isolates were Quellung-nontypeable (0.61%). Of these, 79 (89.8%) contained *cps* loci. Twenty-two nontypeables exhibited serotype 8 *cps* loci with defects, primarily within *wchA*. Six of the remaining nine isolates contained previously-described *aliB* homologs in place of *cps* loci. Multilocus sequence typing revealed that most nontypeables that lacked capsular biosynthetic genes were related to established non-encapsulated lineages. Thus, invasive pneumococcal disease caused by nontypeable pneumococcus remains rare in the United States, and while carriage nontypeables lacking *cps* loci are frequently isolated, such nontypeable are extremely rare in invasive pneumococcal disease. Most invasive nontypeable pneumococci possess defective *cps* locus genes, with an over-representation of defective serotype 8 *cps* variants.

## Introduction

Pneumococcal infections remain a leading cause of morbidity and mortality worldwide [1]. Clinically-used pneumococcal vaccines target the capsular polysaccharide (CPS), which is considered a requirement for virulence and exceeds 94 known serotypes [2]–[6]. Since the implementation of widespread vaccination, serotypes represented in vaccines are isolated less frequently in both nasopharyngeal carriage and invasive pneumococcal disease (IPD), while certain non-vaccine serotypes are isolated more frequently [7]–[9]. Monitoring the serotype distribution of pneumococcal infections is thus vital to the prospective control and prevention of pneumococcal disease.

While the majority of IPD isolates are encapsulated organisms that are serotypeable with typing antisera, nontypeable isolates (NTs) are sometimes recovered [10]–[12]. NTs may represent pneumococci that exhibit novel CPS types, have damaged or substituted capsule synthetic genes, or produce CPS below detection. NTs have been broadly categorized according to the contents of the CPS biosynthetic locus (*cps*), the site of genes for CPS synthesis between *dexB* and *aliA* in all known serotypes but one [2], [13]. Group I NTs retain at least partial *cps* loci; Group II NTs lack *cps* loci but in their place contain genes encoding novel proteins [10], [14]. Park, et al, further divided Group II NTs into clades [14]. Null Capsule Clade (NCC) 1 isolates contain *pspK*, which encodes a novel surface protein [14], [15]. NCC2 and NCC3 isolates contain *aliB* homologs, *aliC* (NCC2 only) and *aliD*, which encode putative lipoproteins of unknown function [10], [12], [14].

NCC isolates have been frequently described among carriage NTs [10], [12], [14]. Because nasopharyngeal carriage of pneumococcus is a prerequisite for IPD, characteristics of carriage isolates have been used to infer characteristics of IPD isolates; however, the nature of IPD NTs is unclear given the protective role of CPS, and the prevalence of IPD NTs is unknown. Rates as high as 3–4% of IPD strains have been reported to be NT based on studies of a limited number of isolates (<30) from Native American populations [11], [16], [17]. However, over 2006 and 2007, NT isolates accounted for only 0.86% of IPD cases reported to the Centers for Disease Control and Prevention through the Active Bacterial Core surveillance program [18]. The majority of characterized IPD NTs have been Group I NT [11], [12], and Scott and colleagues hypothesized that in Group I NTs with intact genetic elements (as determined by microarray), CPS is expressed below what Quellung serotyping would detect [11]. The power of studies on IPD NTs has been limited by the relative rarity of such isolates and lack of detailed sequence analyses of *cps* loci.

From 2006 to 2009, 14,328 pneumococcal IPD isolates were recovered from 10-state population surveillance by Centers for Disease Control and Prevention (CDC) Active Bacterial Core surveillance (ABCs) and subjected to Quellung-based serotyping. Here we describe the characterization of 88 NT strains, the largest collection of IPD NTs examined to date. While the majority were Group I NT, nine Group II NT were identified and analyzed by multi-locus sequence typing (MLST) to determine their relatedness to other NT isolates. In addition, we characterized the molecular defects of the overrepresented PCR-serotype 8 (PCR-8) isolates and determined that all have mutations likely to eliminate CPS production.

## Methods

### Strains and Conditions

Strains were obtained by the CDC ABCs program and cultured in Todd Hewitt broth (BD Biosciences) supplemented with 0.5% yeast extract or on tryptic soy agar plates supplemented with 5% sheep's blood (BAP).

### Quellung Serotyping

Serotyping was performed as previously described [19] by the (*Streptococcus* Laboratory or by the Minnesota Department of Public Health.

### Genetic Analyses

Primers used in this study are presented in Table S1. The CDC PCR-serotyping assays have been described previously [19], [20], updates provided at www.cdc.gov/ncidod/biotech/strep/pcr.htm. One isolate was typed using a separate multiplexed PCR assay, described previously [21]. *cpsA* was detected using multiplex PCR assay [21] and/or in conventional PCR using primer sets cpsA-f/cpsA-r, 5202/3202, or 5694/3694.

Chromosomal DNA from PCR-8 strains was initially assessed for gross defects in *wchA* using primer set 5114/3695, and products were sequenced with primer 5114. For extreme 3′ coverage of *wchA*, amplicons were generated using primer set 5830/3828 and sequenced with primer 5832. For whole *cps* locus sequencing, locus fragments were generated using primer sets 5825/3826, 5830/3828, 5835/3830, and 5840/3832 and sequenced using primers internal to each fragment. PCR using various combinations of primers and agarose gel electrophoresis were used to identify the region of gross defect in isolate 6265-07 prior to sequencing.

Isolate 7236-07 *cps* locus was initially sequenced with primer set 5419/3419 and in subsequent steps by primers generated to sequences from the previous reaction.

Detection of NCC genes [14], PCR for *dexB/aliA* [10], [14], and MLST [22], [23] were performed as previously described.

### DNA Sequencing

PCR products were sequenced by the Heflin Center Genomics Core Lab, University of Alabama at Birmingham.

### Statistical Analyses

Statistical analyses were performed in Microsoft Excel. Testing for overrepresentation among PCR-serotypeable NTs was performed by chi-squared analysis using expected values generated from prevalence data over the study period using an assumed sample size of 79 (i.e., the number of PCR-serotypeable isolates).

Comparisons for PCR-serotypeable isolates relative to Quellung-typeable counterparts were performed by dividing the raw numbers of each group for a given serotype and confined to PCR-serotypes with two or more isolates. Serotype 8 was a statistical outlier as determined by Grubbs' test (http://graphpad.com/quickcalcs/Grubbs1.cfm) and excluded from confidence interval (CI) calculation. CI was calculated manually using standard error and the “tdist” function in Microsoft Excel.

### Nucleotide Sequence Deposition

The *cps* locus sequence of isolate 7236-07 has been deposited at GenBank (accession number KJ363164).

## Results

### Some Invasive Pneumococcal Isolates are Nontypeable

During the period of 2006–2009, the CDC received 14,328 independent IPD isolates. A subset of these isolates (N = 88, 0.61%) was nontypeable by Quellung and subsequently serotyped by PCR. Seventy-nine isolates (89.8% of NT) were PCR-serotypeable or *cpsA*-positive and thus classified as Group I NT; their assigned serotypes, prevalence, and the prevalence of corresponding Quellung-typeable isolates are presented in Table 1. The remainder (10.2%) were Group II nontypeables. Group II NT organisms were thus extremely rare causes of IPD over this period, accounting for only 0.056% of IPD cases.

**Table 1:** PCR-serotype Distribution of Nontypeable Pneumococci, 2006–2009.

PCR serotype or serogroup	Quellung NTs Count *% total Quellung-NT* (N = 88)	Corresponding Quellung Serotype(s) Serotype (count); *% total surveillance isolates* (N = 14,328)
*Group I NT*		
8	22; *25.0*	8 (311); *2.17*
19A	8; *9.09*	19A (2643); *18.4*
7F/7A	7; *7.95*	7F (2006), 7A (1); *14.0*
9N/9L	6; *6.82*	9N (339); *2.37*
22F/22A	4; *4.55*	22F (1000), 22A (1); *6.99*
11A/11D	3; *3.41*	11A (364); *2.54*
16F	3; *3.41*	16F (405); *2.83*
3	2; *2.27*	3 (1139); *7.95*
15B/15C	2; *2.27*	15B (145),15C (145); *2.02*
19F	2; *2.27*	19F (148); *1.03*
23B	2; *2.27*	23B (214); *1.49*
33F/33A/37	2; *2.27*	33F (477), 37(13), 33A (2); *3.43*
34	2; *2.27*	34 (67); *0.47*
4	1; *1.14*	4 (292); *2.04*
6A	1; *1.14*	6A (224); *1.56*
6C/6D	1; *1.14*	6C/6D (651); *4.54*
10A	1; *1.14*	10A (206); *1.44*
15A/15F	1; *1.14*	15A (408), 5F (2); *2.86*
17F	1; *1.14*	17F (179); *1.25*
18C/18B/18A/18F	1; *1.14*	18C (67), 18B (6), 18A (4), 18F (3); *0.56*
23F	1; *1.14*	23F (71); *0.50*
24F/24A/24B	1; *1.14*	24F (3), 24A (1); *0.03*
31	1; *1.14*	31 (242); *1.69*
35F	1; *1.14*	35F (133); *0.93*
*cpsA* ^+^ but not serotypeable by PCR	3; *3.41*	– (–)
*Group II NT*		
NCC2 (*aliC^+^ aliD^+^*)	6; *6.82*	
NCC4 (no ORFs)	1; *1.14*	
Undetermined	2; *2.27*	

### Group II Nontypeable Organisms Are Generally Null Capsule Clade 2

**Figure 1:**
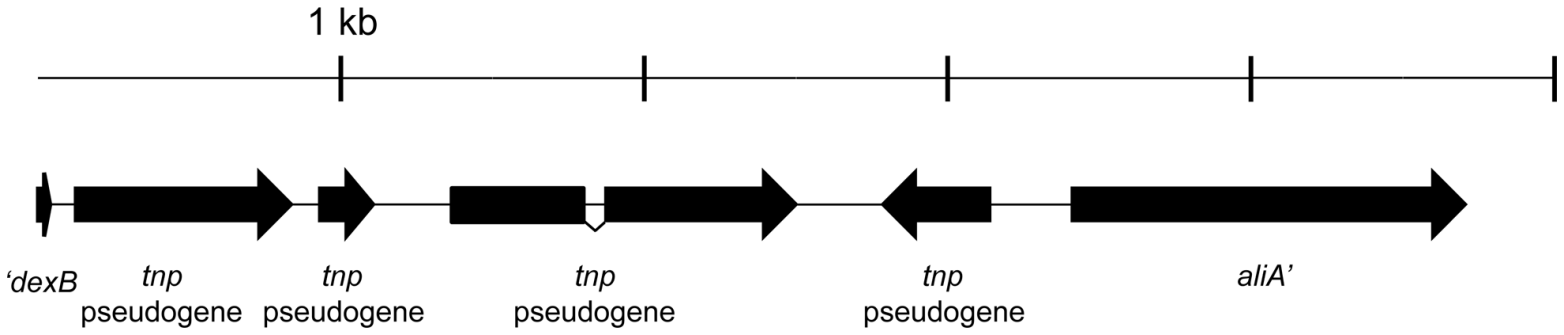
The isolate 7236-07 *cps* locus does not contain capsular biosynthetic or novel surface protein genes. Schematic of the *cps* locus between primers 5419 and 3419 from isolate 7236-07 (deposited to GenBank, accession no. KJ363164). Genes and spacing are to scale. *dexB* and *aliA* are truncated at their 5′ and 3′ ends, respectively, in the sequence coverage. Annotations of *tnp* pseudogenes were assigned corresponding to the serotype 5 *cps* locus (GenBank no. CR931637).

The Group II isolates were assayed by PCR for the presence of NCC-associated genes (i.e., *pspK*, *aliC*, and *aliD*) [14]. No isolates exhibiting *pspK* (NCC1) or *aliD* only (NCC3) were identified; however, six isolates contained both *aliC* and *aliD* and were classified as NCC2 (Table 1). The *cps* locus arrangements of NCC1, NCC2, and NCC3 have been described in detail by others [10], [12], [14]. Chromosomal DNA from the remaining three isolates was subjected to PCR using primers spanning *dexB* and *aliA* [10], [14].Two of these isolates repeatedly failed to produce a product; however, isolate 7236-07 demonstrated a product ∼5 kb in length. We sequenced isolate 7236-07 through this region and determined that it contained only transposon-related elements between *dexB* and *aliA* (Fig. 1, sequence submitted to GenBank, accession no. KJ363164) and was 99% identical to the transposon elements downstream of the serotype 5 *cps* locus over their shared sequence (accession no. CR931637, [2]). This is the third IPD isolate to be described with only transposable elements in its *cps* locus [12], and we propose that NCC4 be defined as Group II NT isolates lacking non-transposon open reading frames [10], [14], [15].

### Group II Nontypeables Obtained in Surveillance Are Related to Established Nontypeable Lineages

Because these Group II NT isolates were obtained from patients with IPD rather than nasopharyngeal carriage, we used MLST to assess their relatedness to previously described nontypeable lineages (Table 2). Four of these strains revealed MLST genotypes identical (ST344, ST448, and ST1186) or similar (ST1540, sharing 5 of 7 alleles with ST344) to those associated with previously-reported conjunctivitis outbreaks [24], [25]. ST1540 was previously recorded from a nontypeable carriage isolate according to the MLST database (http://www.mlst.net). Isolate 7236-07 is the first NCC4 strain to our knowledge in which sufficient residual elements remain to facilitate the identification of the likely ancestral serotype, in this case, serotype 5; consistent with this observation, the sequence type of this strain, ST4840, was associated with two serotype 5 isolates (www.mlst.net).

**Table 2:** Multi-locus sequence types of Group II Nontypeable Isolates, 2006–2009.

Isolate	ST^1^	Related Serotype(s)	Comments
*NCC2 (aliC^+^ aliD^+^)*			
2008236031	448	NT	Associated with conjunctivitis
4670-07	1054	NT	Two-locus variant of ST344
9034-06	344	NT	Associated with conjunctivitis
8542-07	1186	NT	Associated with conjunctivitis
2010216117	1540	NT, 15	Double locus variant of ST4589 (associated with NT and 9V)
2010201915	1540	NT, 15	(*see above*)
*NCC4 (no known ORFs)*			
7236-07	4840	5	Serotype 5 lineage
*Not Determined*			
8296-06	1390	6A and 6C	
2008230408	191	7F and NT	NTs represent 7 of 274 reported ST191 isolates

1 Data obtained from MLST.net, accessed January 26, 2014.

Of the two isolates failing to produce a PCR product in the *dexB/aliA* region, one belonged to ST191, which has been rarely reported in association with NT organisms but is strongly associated with serotype 7F; the remaining isolate belonged to ST1390, which is associated with serotypes 6A and 6C (Table 2). Because these two isolates were *cpsA*-negative and share STs associated with serotypes detectable in our PCR assays, it is likely that these strains exhibit gross defects around their respective *cps* loci.

### PCR-8 Nontypeables Are Overrepresented in Group I Nontypeables and Contain Inactivating Mutations in wchA

The serotypes identified by multiplex PCR are listed in Table 1. When compared to the prevalence of encapsulated serotype 8 isolates, PCR-8 NTs were significantly overrepresented among NT isolates (*P*<<0.001). Five additional NT isolates obtained in mixed culture with Quellung- serotype 8 isolates were determined to be PCR-8 and were also examined (Table 3). Identical antibiotic susceptibilities were obtained for these isolates and their Quellung counterparts, suggesting that the NTs were derived from the encapsulated isolates (data not shown).

**Table 3:** Mutations in PCR-serotype 8 Isolates.

Isolate	Mutation(s)^a^^,^ b	Result^c^
*Isolates obtained as nontypeable in pure culture*		
4179-08	T2158G, T5050A, G5230A, G5252A, C5540T	Putative promoter −10 region mutation, Wzd (D226E), Wze (T51T, A59T, P155S)
6265-07	ΔA5217-A8124	Wze (E57FS^d^), Δ*wchA-wciR*
2010200824	G5869T	WchA (E30* d)
7518-07	G5875T	WchA (E32*)
8286-06	ΔC5900	WchA (A40FS)
8199-06	Tandem repeat of 5960-5968	WchA (Y63*)
8390-06	Insert C before C6003	WchA (L75FS)
2008008783	C6084A	WchA (Y101*)
7631-07	ΔA6232-G6619	WchA (I151FS)
9164-06	G6508T	WchA (G243*)
8083-06	C6682T	WchA (R301C)
2008232774	C6717G	WchA (Y312*)
8158-06	A6718G	WchA (K313E)^e^
8402-06	A6718G	WchA (K313E)^e^
2008008362	C6724G	WchA (R315G)
7445-07	ΔA6751	WchA (R326FS)
2009211113	C6784T	WchA (Q335*)
2008008580	G6833A	WchA (G351D)
8174-06	C6844G	WchA (R355G)
7145-06	A6872T	WchA (Q364L)
6841-06	C6920T	WchA (P380L)
2008227824	C6923T	WchA (P381L)
*Nontypeable isolates obtained in mixed culture with a Quellung-positive isolate*		
8992-06	ΔC6194	WchA (A38FS)
5238-07	ΔA6690	WchA (K304FS)
5241-07	C6861A	WchA (D360E)
2009208249	C6923G	WchA (P381R)
2009212775	G7090A	WchA (D437N)

a Sequence numbers correspond to GenBank accession No. AJ239004.

b Polymorphisms present in an alternative serotype 8 sequence (accession No. CR931644) were ignored.

c Residue numbers correspond to amino acid sequence deduced from *cap8C* (Wzd), *cap8D* (Wze), or *cap8E* (WchA) in GenBank accession No. AJ239004.

d FS, frameshift;

*, stop.

e Residue previously identified as a site of inactivating mutation [29].

**Figure 2:**
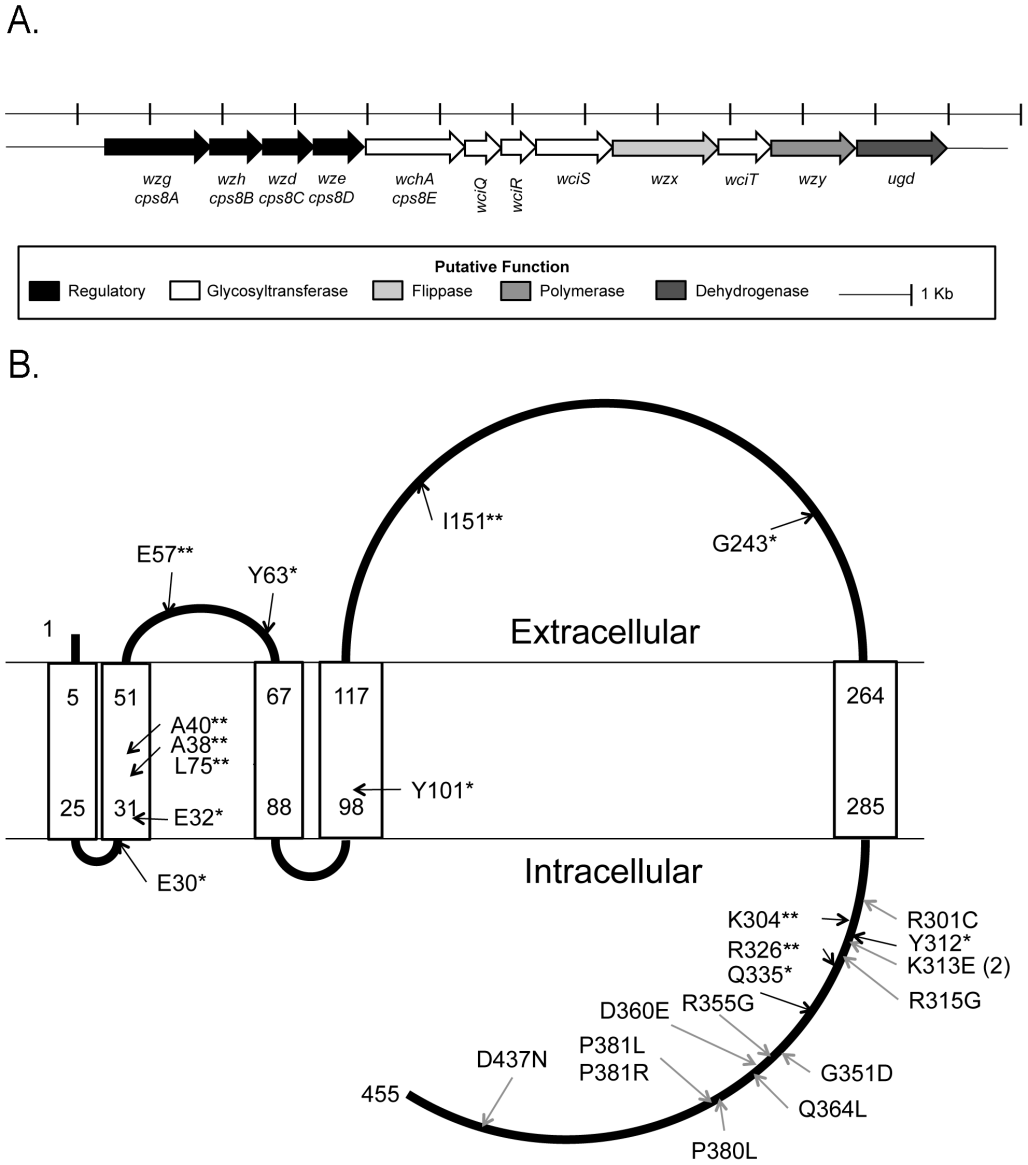
PCR-8 nontypeable isolates contain inactivating mutations in *wchA*. A. Schematic of the serotype 8 *cps* locus (GenBank accession No. AJ239004). Alternative nomenclature is shown for regulatory genes. B. Molecular model of WchA as predicted by TMPRED (http://www.ch.embnet.org/software/TMPRED_form.html). Black arrows indicate nonsense (*) and frameshift (**) mutations; gray arrows indicate missense mutations. Residue numbers correspond to the predicted primary amino acid sequence for WchA.

To investigate whether PCR-8 NTs express minor amounts of capsule, we looked for molecular defects within their *cps* loci. Because loss of CPS production in pneumococci frequently results from mutations in *wchA* [26]–[29], we amplified and sequenced *wchA* in these isolates (the serotype 8 *cps* locus is presented in Figure 2A). When compared to two serotype 8 *cps* locus reference sequences [2], [30], twenty-three of the twenty-seven PCR-8 strains contained single base pair changes in *wchA*, summarized in Table 3 and whose effects are shown in a molecular model of WchA (Figure 2B). Eleven mutations resulted in premature termination codons or frameshifts predicted to truncate WchA before amino acid 335 (Figure 2B). Twelve missense mutations were identified and were confined to the predicted C-terminal loop encoding the catalytic portion of the enzyme (Figure 2B, [31]). Isolate 8199-06 contained a short tandem duplication event, and sequencing revealed a 388-bp deletion internal to *wchA* of isolate 7631-07 (Table 3). The *wchA* mutations were likely primary, as we sequenced the entire *cps* locus from two isolates and found no other genetic defects than the *wchA* mutation. This conclusion is also supported by the findings of Melchiorre, et al., who identified mutations in *wchA* in two serotype 7F IPD NTs and restored CPS in those strains through overexpression of wild-type *wchA* [27].

Because isolate 6265-07 reproducibly failed to produce a *wchA* product, primers outside this region were used to localize the defect, and sequencing revealed a large deletion in the *cps* locus spanning ∼80% of *wze* and all of *wchA, wciQ*, and *wciR* (Table 3, Figure 2A). The final isolate, 4179-08, exhibited no mutation in *wchA* despite producing no CPS as detectable by a flow cytometric serotyping assay (not shown), and its entire *cps* locus was sequenced. We identified five single base pair changes resulting in alterations to the putative *cps* gene promoter, *wzd* (a.k.a. *cap8C, cps8C*), and *wze* (a.k.a. *cap8D, cps8D*)(Table 3, Figure 2A).

## Conclusions/Discussion

Although large numbers of carriage NTs have been studied, only a limited number of IPD NTs have been studied [10]–[12], [27]. Our study shows that they are isolated from patients with IPD in less than 1% of cases. Only a small fraction of IPD NTs (7 of 88) were Group II NTs (i.e., conclusively lacked *cps* loci) [14]. Most such isolates (6 of 7) had *aliB* homologs in place of the *cps* locus, a frequently-described finding in Group II NTs of nasopharyngeal origin [10], [12], [14]. The extremely low prevalence of these strains is not surprising when considering that the acquisition of these novel *cps* locus-replacing genes almost certainly arose as a fitness advantage during colonization, as invasive illness is considered a dead-end path for the organism. Indeed, the recently-identified PspK protein has been shown to increase adherence during colonization [12], [14], [15]. AliB homologs do not appear to influence adherence to epithelial cells but likely provide advantages in other facets of colonization [10]. Because their prevalence in IPD is so rare, NCC isolates may have been obtained from patients who were already immunocompromised or otherwise susceptible to infection.

Consistent with previous studies, we found that the vast majority of these isolates are Group I NTs, retaining at least portions of their *cps* loci [11], but a striking observation is that Group I NTs tend to have serotype 8 *cps* loci. The observed frequency of PCR-8 among NTs vastly exceeds serotype 8 prevalence in IPD. The appearance of acapsular serotype 8 strains under continuous culture conditions using sorbarods has been described; these isolates lost CPS expression due to introduction of a tandem duplication within *wchA* [28]. However, we did not find a fixed pattern in *wchA* mutations, nor did we find any characteristic in serotype 8 *wchA* that should predispose it to mutation. In fact, of the other PCR serogroups/-types identified in this study with *wchA* genes homologous to that of serotype 8 (serotypes 6, 8, 11A, 15A, 17F, 19A, 23F, 23B, 33F, and 34) [2], [32], sharing between 93.0% and 96.5% nucleotide identity (analysis of sequences in references 2 and 30) only serotype 8 was overrepresented among IPD NTs. Only three isolates retain the potential to revert (two potential slip strand mutations and one tandem repeat [28]); however, with at longest a nine bp insertion, the expected frequency of reversion would be at most ∼10^−5^ [28], [33]. The even distribution between frameshift and point mutations in *wchA*, in addition to the observation of similar mutations found during generation of otherwise-lethal mutations in the *cps* locus [26], [29], suggests that these NTs are producing very little to no CPS rather than a down-regulated amount of capsule as has been hypothesized [11]. All mutations in *wchA* are predicted to perturb or eliminate the C-terminal catalytic domain of WchA [31]. We thus conclude that the majority of IPD NTs are defective in CPS production, frequently due to point mutations in *wchA* that would not be identified in superficial genetic analyses such as PCR or microarray.

The inactivation of CPS expression in invasive Group I NTs is unexpected because of the protective role CPS plays for pneumococci. These isolates may thus represent an in vitro artifact and arise through an unknown mechanism during isolation and subculture in the laboratory. Although in certain serotypes (e.g., serotype 3, which exhibits mucoid colonies) nonencapsulated strains are easily distinguished from encapsulated strains on BAP, in other serotypes, the difference between a small colony and a nonencapsulated colony is not always apparent. Because it is common practice to select only one colony from a culture for Quellung analysis, the chance of inadvertently selecting a CPS-negative colony would increase the closer in size it is to the CPS-positive colony, leading to a selection bias. For serotype 3, one would expect a bias in the opposite direction, and this was what was observed: the ratio of PCR-3 isolates to Quellung-serotype 3 (0.0018) was the lowest when compared to this metric for other serotypes with at least 2 NT isolates (mean  = 0.0091; 95% CI 0.0040–0.0142). However, serotypes 34 and 9N/9L were above the CI, and their individual chi-squared values when compared to their expected prevalences (7.14 and 9.17, respectively) were unremarkable as compared to that of serotype 8 (240.05, ratio  = 0.071), so this does not appear to completely explain the abundance of PCR-8 isolates.

Alternatively, one could envision a population of acapsular organisms arising during infection as the host response begins to target CPS. Under such circumstances, encapsulated organisms would be targeted by antibodies or innate lectins and rapidly opsonized with C3b through the classical or lectin pathways, depleting complement levels in a local environment to sufficiently permit transient survival of nonencapsulated organisms, which would still possess pneumococcal anti-complement enzymes (reviewed in reference [34]). This could account for the observation that NT isolates of certain serotypes are sometimes obtained in mixed culture with encapsulated isolates (LK Sherwood and B Beall, unpublished observations). Less extreme microevolutions, involving intra-serogroup changes, are known to take place. For example, in serogroups 9 and 11, one member of the serogroup (9A/11E) appears to arise independently from a different member of the serogroup (9V/11A); in both cases, the serotype switch occurs through inactivation of a single gene in the *cps* locus encoding an acetyl transferase, resulting in absence of an acetylated residue on the CPS repeat unit [35], [36]. Because each isolate of 9A/11E contained a different inactivating mutation, the authors concluded that these serotypes have arisen repeatedly over time during infection from strains producing the acetylated version of the CPS [35]–[37]. The low prevalence of IPD NTs could suggest underlying defects in the host patient's immunity, permitting survival of an unencapsulated population during infection.

In contrast to carriage, where Group II NTs are frequently isolated [10], [12], [14], IPD NTs are composed primarily of Group I NTs with defective *cps* loci rather than merely reduced CPS expression. Regardless of how Group I IPD NTs arise, this finding reaffirms the essential nature of CPS in at minimum the establishment of IPD and suggests that the rare Group II IPD NT infection is likely to occur in an individual predisposed to such infections due to underlying immune defect. This finding, taken with recent work showing microevolution of CPS serotypes—likely occurring within the host during IPD [35]–[37]—suggests that caution is necessary when inferring characteristics of invasive isolates based on those determined of carriage strains.

## Supporting Information

Table S1 **Primers used in this study.**
(DOCX)
