# Supplementary material for: Population-Based Analysis of Invasive Nontypeable Pneumococci Reveals That Most Have Defective Capsule Synthesis Genes
Source: PLoS One. 2014 May 15;9(5):e97825. doi: 10.1371/journal.pone.0097825 (PMC4022640; doi:10.1371/journal.pone.0097825)
Supplement: Table S1 — Primers used in this study. (DOCX) [file pone.0097825.s001.docx]

**Table S1. Primers used in this study.**

| **Primer** | **Sequence (5'-3')^a^** | **Source^b^** |
| --- | --- | --- |
| *Forward Primers* | |  |
| 5114 | TTAGTGACGGAGGCAGGTGAA | [[1](#_ENREF_1)] |
| 5202 | CAACCGTACAGAATGAAGCGG | [[2](#_ENREF_2)] |
| 5419 | TCTTAGTTCCATGGGATGCTTTCTGTGTG | *dexB,* [[3](#_ENREF_3)] |
| 5511 | AACCTCAACTATTTGACAACTGCTA | 7236-07^3553-3577^ |
| 5694 | GGGAACGACTATCCTGTTGGAAATC | [[4](#_ENREF_4)] |
| 5815 | GAGGACTTTCTTTTTAGTATAATAAAGTTAG | 7236-07^872-902^ |
| 5816 | TATGCGATAGCAGTCCAAGG | 7236-07^1836-1855^ |
| 5817 | ACGCTTTTCTTCCAGGTGG | 7236-07^2805-2823^ |
| 5825 | GATGCAGATAGTGAAAAAAAAGGTG | ST8^2105-2129^ |
| 5826 | CATCGAGTTAGAGTATCCAGAC | ST8^2761-2782^ |
| 5827 | GATTTAGTGAATACTCAGTTGGAAAG | ST8^3446-3471^ |
| 5828 | CAAGTAAATAGTTCACATGTCCTC | ST8^4116-4139^ |
| 5829 | CCAGCAAAAACGTTAGCTAGC | ST8^4685-4705^ |
| 5830 | CGTTCCAGATTTGGACAAAATG | ST8^5041-5062^ |
| 5831 | CAGTAAACAAATACGGTTCTTATGG | ST8^5709-5733^ |
| 5832 | GAGAGCTTGTCTCTCAGTTTG | ST8^6404-6424^ |
| 5833 | GGACAATCTGGAAAGATATTGAAATC | ST8^7076-7101^ |
| 5834 | GTGTTTGCTCAGATTGGAGC | ST8^7730-7749^ |
| 5835 | CGACAATACATAGATAGTCTTTG | ST8^8090-8112^ |
| 5836 | GCATTTGACAATGGCTGGAG | ST8^8743-8762^ |
| 5837 | GTCAGACTGTCAGAGAATTGTTAG | ST8^9428-9451^ |
| 5838 | ATTGCGGGTAGGAATTACTTAG | ST8^10135-10156^ |
| 5839 | GGAAATGGGCATTTGCATCG | ST8^10838-10857^ |
| 5840 | GAGGAAAATAACCAATGCCGTATC | ST8^11361-11384^ |
| 5841 | GCTCTACTAGTATCTATAGTAATTTTTG | ST8^11996-12023^ |
| 5842 | CATTGCGGTTTTATTGGCTCAG | ST8^12648-12669^ |
| 5843 | CTCAAGCTATTATTGATGGAGTTGG | ST8^13298-13322^ |
| cpsA-f | GCAGTACAGCAGTTTGTTGGACTGACC | [[2](#_ENREF_2)] |
| *Reverse Primers* | |  |
| 3202 | GAATATTTTCATTATCAGTCCCAGTC | [[2](#_ENREF_2),[4](#_ENREF_4)] |
| 3419 | CGCTGAACTTTTGTAGTTGCTGTCTGGTCAAC | *aliA*, [[3](#_ENREF_3)] |
| 3694 | CTTCTGTTGATTCCGTCCTCGATC | [[4](#_ENREF_4)] |
| 3695 | CTTAATCTCACTTCGTCCACTGACC | ST8^7029-7005^ |
| 3753 | TTGGTTTTCGGAACTTTGAAAACTTC | 7236-07^245-220^ |
| 3754 | ACTTGTTCACGAAGAAGGGC | 7236-07^1004-985^ |
| 3755 | TCACATGCGCCCAACATC | 7236-07^1955-1938^ |
| 3756 | CTCAAATTGTCTAGTCTCTATGC | 7236-07^2918-2896^ |
| 3817 | CTGTAAGGCTACCATCTTTAAAGTTTTC | 7236-07^4233-4206^ |
| 3825 | GTCTGGATACTCTAACTCGATG | ST8^2782-2761^ |
| 3826 | CTAATGTTGGCATAACTTCCTC | ST8^5090-5069^ |
| 3827 | CCATAAGAACCGTATTTGTTTACTG | ST8^5733-5709^ |
| 3828 | GCAAAACTTTTGTCATCTAAATTTCC | ST8^8141-8116^ |
| 3829 | CTCCAGCCATTGTCAAATGC | ST8^8762-8743^ |
| 3830 | CAAATAATCTTATAGAAAATTTATTTTGCGG | ST8^11426-11396^ |
| 3831 | CAAAAATTACTATAGATACTAGTAGAGC | ST8^12023-11996^ |
| 3832 | TTCTCAGTATAGACCGCTTCTAAC | ST8^13900-13877^ |
| cpsA-R | GAATATTTTCATTATCAGTCCCAGTC | [[2](#_ENREF_2)] |

^a^Underlined base indicates departure from reference sequence

^b^Nucleotide references correspond to GenBank accession Nos. AJ239004 (ST8) or KJ363164 (7236-07)

**References**

1. Bratcher PE, Park IH, Hollingshead SK, Nahm MH (2009) Production of a unique pneumococcal capsule serotype belonging to serogroup 6. Microbiology 155: 576-583.

2. Pai R, Gertz RE, Beall B (2006) Sequential multiplex PCR approach for determining capsular serotypes of *Streptococcus pneumoniae* isolates. J Clin Microbiol 44: 124-131.

3. Hathaway LJ, Stutzmann Meier P, Battig P, Aebi S, Muhlemann K (2004) A homologue of *aliB* is found in the capsule region of nonencapsulated *Streptococcus pneumoniae*. J Bacteriol 186: 3721-3729.

4. Yu J, Lin J, Benjamin WH, Jr., Waites KB, Lee CH, et al. (2005) Rapid multiplex assay for serotyping pneumococci with monoclonal and polyclonal antibodies. J Clin Microbiol 43: 156-162.
